# Knowledge, Attitude, and Practice towards Labor Pain Management and Associated Factors among Skilled Birth Attendants Working at Hospitals Found in Central, West, and North Gondar Zones, Northwest Ethiopia, 2019: A Multicenter Cross-Sectional Study

**DOI:** 10.1155/2021/8835677

**Published:** 2021-05-13

**Authors:** Eyasu Tekile Solomon, Fisseha Yetwale Kassie, Dawit Gebeyehu Mekonnen, Muhabaw Shumye Mihret, Addisu Taye Abate, Amanuel Addisu Dessie

**Affiliations:** ^1^Amhara Regional Health Bureau, Amhara National Regional State, Bahir Dar, Ethiopia; ^2^Department of General Midwifery, School of Midwifery, College of Medicine and Health Sciences, University of Gondar, Gondar, Ethiopia; ^3^Department of Clinical Midwifery, School of Midwifery, College of Medicine and Health Sciences, University of Gondar, Gondar, Ethiopia; ^4^School of Nursing, College of Medicine and Health Sciences, University of Gondar, Gondar, Ethiopia; ^5^Department of Public Health, College of Health Sciences, Woldia University, Woldia, Ethiopia

## Abstract

**Introduction:**

Delivery of the infant into the arms of a conscious and pain-free mother is the most exciting and rewarding moment in maternal care services. Physical and mental care of women during delivery requires good knowledge and a positive insight to the needs and rights of the mothers. Little was known regarding skilled birth attendants' knowledge, attitude, and practice towards labor pain management in the study area. Hence, the current study aimed at assessing knowledge, attitude, and practice, and associated factors towards labor pain management among skilled birth attendants working at hospitals found in central, west, and north Gondar zones, northwest Ethiopia, 2019.

**Method:**

A multicenter institution-based cross-sectional study was conducted from June 1 to 30, 2019. A census sampling technique was used to include a total of 336 skill birth attendants. A pretested standardized self-administered questionnaire was used to collect the data. The data were then entered into Epi Info 7.1.2 and exported to SPSS version 25 for analysis. Multivariable logistic regression analyses were undertaken to identify factors associated with outcome variables. The level of significance of the study was declared based on adjusted odds ratio with 95% confidence interval at a *p* value of ≤0.05.

**Result:**

The proportion of skill birth attendants having good knowledge, a favorable attitude, and a good practice on labor pain relief methods was 47%, 41.96%, and 57.14%, respectively. Age of ≤30 years (AOR = 5.43; 95% CI: 1.25, 23.53), educational status of 2^nd^ degree and above (AOR = 3.56; 95% CI: 1.32, 9.60), working at a private primary hospital (AOR: = 6.55; 95% CI: 2.15, 19.93), and working at a referral hospital (AOR = 2.24 : 95% CI: 1.01, 4.93) are factors significantly associated with good knowledge while having good knowledge on labor pain relief methods (AOR = 2.26; 95% CI: 1.42, 3.60) and working at private primary hospitals (AOR = 7.01; 95% CI: 1.92, 25.65) had statistically significant association with favorable attitude and good practice on labor pain relief methods, respectively. *Conclusion and Recommendations*. Poor knowledge, unfavorable attitude, and poor practice towards labor pain management were found in this study. Strengthening the capacity of public health facilities and providing continuous professional development (CPD) training for the skilled birth attendants would be helpful in improving knowledge, attitude, and practice towards labor pain management.

## 1. Introduction

For almost all women, labor involves pain which is often described as the most severe pain that a woman ever has to face, and it is caused by uterine contractions, associated with dilation of the cervix and stretching of the lower uterine segment [1]. Pain management during child birth is the treatment or prevention of pain that a woman may experience during labor and delivery, by using pharmacological options, oral tablets, inhalation analgesia, intravenous or intramuscular opioids and regional analgesia and/or nonpharmacological options including continuous support of a companion, directed breathing and relaxation techniques, massage, laboring in water, and the use of transcutaneous electrical nerve stimulation in early labor [2, 3].

Ensuring births assisted by skilled birth attendants (SBAs) is a crucial approach in reducing maternal and newborn morbidity and mortality [4]. However, evidence suggests that there is a high disparity in proportion of laboring mothers attended by SBAs across sociodemographic status of the women. For example, approximately 72% of births among rural mothers were attended by SBAs while that of among urban resident mothers was about 90% globally in 2019 [4]. In Ethiopia, about 80% and 21% of the births were attended by skilled birth attendants in urban and rural-based health facilities, respectively [4].

One of the most exciting and rewarding moments for all SBAs is assisting the birth of an infant with a pain-free mother [5, 6]. The American College of Obstetricians and Gynecologists (ACOG) describes that there is no other circumstance where it is considered as acceptable for a laboring mother to experience untreated severe pain during the event of giving birth, while under a physician's care [7]. Thus, pain relief in labor is one of the basic competencies of SBAs and considered as an essential part of intrapartum care. In this context, all women have the need to receive a range of various pain relief options for labor and delivery. In addition, providing good pain relief options during labor could be a critical incentive to increase healthcare facility deliveries since it motivates and encourages mothers to have facility-based deliveries rather than home-based deliveries by traditional birth attendants, and in turn may ultimately decrease the prevalence of labor and delivery related maternal morbidities and mortalities. Despite this fact, many of the laboring women suffer from unmanaged labor pain that can be subsided through quality and compassionate pain management [3] and the worst scenario in this regard happens in developing countries including Ethiopia where most women still go through a painful labor [5, 6].

Ethiopian government has strived to enhance provision of physical, psychological, and pharmacological methods of labor pain relief methods and put it as core competencies under practice standard III of Ethiopian Federal Ministry of Health (EFMoH) [8]. However, provision of both pharmacologic and nonpharmacologic pain relief options for labor remains infrequent [3, 9]. This may be as a result of several factors, including lack of awareness, healthcare delivery systems, misunderstanding regarding acceptability, and safety and availability of pain relief options. In this perspective, growing the knowledge and attitude of SBAs towards labor pain management is paramount [10, 11].

Therefore, evidence in this regard is compulsory. However, there was scarce of shreds of evidence about the knowledge, attitude, and practice and predictors regarding labor pain management among SBAs working in Ethiopia. This study, therefore, aimed at assessing knowledge, attitude, and practice on labor pain management and associated factors among SBAs working at hospitals found in central, west, and north Gondar zones, northwest Ethiopia, 2019.

We hypothesize the following:

1. Skilled birth attendants' knowledge of labor pain management (LPM) can be significantly associated with their sociodemographic characteristics, education-related factors, and institution-related factors such as type of hospital.
2. Skilled birth attendants' attitudes towards LPM can be influenced by their sociodemographic characteristics, education-related factors, knowledge on LPM, and institution-related factors such as type of a hospital.
3. The level of LPM practice by SBAs can be affected by various factors such as the practitioners' sociodemographic characteristics, education-related factors, knowledge status on LPM, attitude towards LPM, and institution-related factors such as the type of hospital.

## 2. Methods

### 2.1. Study Design, Period, and Settings

An institution-based cross-sectional study was conducted from June 1^st^ to 30^th^, 2019. The study was conducted in all hospitals found in central, west, and north Gondar zones which are located in the Amhara National Region of Ethiopia. In the three zones, there are a total of 14 hospitals including 11 primary public hospitals, one specialized public hospital, and 2 private hospitals which provide services for over 7 million people from the surrounding area. In all hospitals, labor and delivery services are provided.

### 2.2. Source and Study Population

All SBAs working at hospitals found in the central, west, and north Gondar zones were the source population while those SBAs who were working at labor and delivery wards of the hospitals during the study period were the study population.

### 2.3. Inclusion and Exclusion Criteria

All permanently employed SBAs who were working at labor and delivery wards of the hospitals during the study period were included in the study, whereas those SBAs who were not available (i.e., who were on annual leave, maternity leave, or seriously ill) during the time of data collection were excluded from the study.

### 2.4. Sample Size Determination, Sampling Technique, and Procedures

The sample size was estimated by using single population proportion formula with the following assumptions: proportion of SBAs having good knowledge, favorable attitude, and good practice on LPM −60.1%, 56.7%, and 43.3%, respectively, based on a study done in northern Ethiopia [12], level of confidence 95%, margin of error 5%, and nonresponse rate 10%. Thus, the sample size (*n*) was calculated as follows: *n* = [(*Z α*/2)^2^^*∗*^*P*(*q*)]/*d*^2^; where *n* = sample size, *Zα*/2 = 1.96, *d* = margin of error (5%), *p* = prevalence in previous study (*P* = 60.1 % = 0.601 for knowledge, *p* = 56.7% = 0.567 for attitude and *P* = 43.3% = 0.433 for practice), *q* = 1−*P* (i.e., *q* = 0.399 for knowledge, 0.433 for attitude, and 0.567 for practice). Accordingly, the sample size (*n*) = [(1.96)^2^^*∗*^ 0.601 ^*∗*^ (1–0.601)]/(0.05)^2^ = 369 for knowledge, [(1.96)^2^^*∗*^ 0.567 ^*∗*^ (1–0.567)]/(0.05)^2^ = 378 for attitude and [(1.96)^2^^*∗*^ 0.433 ^*∗*^ (1–0.433)]/(0.05)2 = 378 for practice. After taking the largest one (i.e., *n* = 378) and adding 10% nonresponse rate, the final total sample size becomes 417. However, since the actual number of the study population was less than the calculated sample size, all (363) SBAs were included in the study by using census sampling method.

### 2.5. Study Variables

The dependent variables of this study include knowledge on LPM, attitude towards LPM, and practice towards LPM, whereas the explanatory variables include sociodemographic factors (such as age, sex, religion, monthly income, and marital status), education-related factors (like level of education, profession, years of experience, and in-service training on labor pain management), and institution-related factors (health institution policy, number of SBAs, and availability of equipment and analgesic drugs).

### 2.6. Operational and Term definitions

  Skilled birth attendant: an accredited health professional such as a midwife, doctor, health officer, or nurse who has been educated and trained to proficiency in the skill needed to manage normal pregnancies, childbirth, and the immediate postnatal period, and in identification, management, and referral of complications in women and newborns
  Good knowledge: those who scored mean value and above on knowledge-related LPM questions
  Favorable (positive) attitude: those who scored mean value and above on attitude-related LPM questions
  Good practice: those who scored mean value of and above on practice-related LPM *t* questions

### 2.7. Data Collection Methods, Tools, and Procedures

The source of data for this study was based on the self-report of respondents with no objective measures (like observation) to evaluate them. Data were collected by using a pretested and structured self-administered questionnaire. The questionnaire has four parts: the first section of the questionnaire contains *s* sociodemographic factors (such as age, sex, religion, and marital status), education-related factors (such as level of education, profession, and professional experience), and institution-related factors (such as health institution policy, availability of equipment, and analgesic drugs). The second section contains knowledge-related questions. The third section has attitude-related questions and the fourth section comprises practice-related questions (Additional file 1). The tool was adopted from a previous related study [13]. A total of seven midwives with diploma level of education were employed as data collectors and 3 BSc midwives who had an experience of data collection were employed as supervisors. After the study purpose was briefly presented and informed consent was taken from each study participant, the data collectors distributed the questionnaire and collected the data.

### 2.8. Data Quality Control

The quality of data was ensured by proper designing and pretesting of the questionnaire at Adiss Zemen and Debre Tabor Hospitals on 42 SBAs prior to the actual data collection to ensure quality, clarity, and understandability of the questionnaire. Based on the pretest feedback, some questions were rephrased and the final questionnaire was prepared. Training was given to the data collectors and supervisors for one full day regarding the questionnaire (on the objective of the study, how to approach the study participants, and how to administer and collect the questionnaires timely). The respondents were given brief orientation before they started to fill the questionnaire. Every day after the data collection, filled questionnaires were reviewed and checked for completeness and relevance by the supervisors and the principal investigator. For controlling errors during data analysis, frequency check was done for each variable.

### 2.9. Data Processing and Analysis

All the questionnaires had been checked visually and coded and the data were entered into EPI Info version 7 and then exported to SPSS Version 22 for analysis. Data analysis was carried out using SPSS version 22 software. Descriptive statistics such as frequencies mean and median with standard deviation had been calculated and illustrated by using tables, graphs, and charts. Binary logistic regression was run to see the crude significant relation of each independent variable with the dependent variables. Variables with *p* value less than or equal to 0.20 were fitted into multivariable logistic regressions for controlling the possible effect of confounders, and finally the variables which had independent association with LPM were identified on the basis of AOR, with 95% CI and *p* value less than 0.05.

## 3. Results

### 3.1. Sociodemographic Characteristics of the Respondents

From the total of 363 study participants required for the study, only 336 participated and filled the questionnaire, giving a total response rate of 92.56%. The mean age of the participants was 27.6, and the minimum age recorded was 22 years, while 38 years was the maximum age. More than half (58%) of the participants were males and the majority (64%) of the respondents were Orthodox Tewahido religion followers. Looking at the profession of the participants, more than half (56%) of them were midwives A significant difference was observed on the level of education of the participants and on their working institution, where almost 90% of them were first-degree holders and just above 82% of the respondents were working in public primary hospitals. Almost all (98.5%) of the participants had confessed that at least one labor analgesia was available in their working institution (Table 1).

### 3.2. Knowledge about LPM

The proportion of SBAs having good knowledge on LPRMs was 47.02% (95% CI: 41.4%, 52.4%).

Moreover, 60 (17.9%) of the respondents had reported that they knew only pharmacological LPRMs while 50 (14.9%) of the participants had reported that they knew only nonpharmacological methods and 225 (67.0%) of the respondents reported that they knew both methods. Among pharmacologic methods known by the study participants, about 238 (70.8%) knew steroidal drugs, 145 (43.2%) knew systemic opioids, 77 (22.9%) knew epidural analgesia, and 20 (6%) knew inhalational methods, respectively.

Among the nonpharmacologic LPRMs mentioned by the respondents, providing psychotherapy, allowing the mother to ambulate, massaging the back, allowing free vertical positioning, and showing the woman how to bear down had accounted for the majority of the respondents' response (Table 2).

### 3.3. Attitude of SBAs towards LPM

About 141 (41.96%) of the SBAs had a favorable attitude towards LPM (95% CI: 36.9%, 47.9%).

### 3.4. Practice of LPM

This study relieved that 192 (57.14%) of the study participants had a good practice of LPM (95% CI: 51.8, 62.2).

In this study, in the past one month prior to the data collection, about 14.34% of the respondents had confessed that they provided at least one of the pharmacological LPRMs, 20.72% of the respondents confessed that they provided at least one of the nonpharmacological methods, and the majority (64.94%) of them reported that they provided at least one in both of the methods.

Among the pharmacological LPRMs, pethidine (48.37%), paracetamol (31.37%), and diclofenac (20.26%) were provided by the respondents in the last one month prior to the data collection period. Concerning the nonpharmacological LPRMs provision in the preceding one month prior to the data collection period; about 23%, 20.16%, 18.39%, 17.98%, and 17.03% of the SBAs showed the woman how to bear down, massaged the woman's back, allowed mothers to ambulate, gave mothers psychotherapy, and allowed free vertical positioning, respectively.

### 3.5. Factors Associated with Knowledge of SBA on LPM

Sex, age, level of education, type of hospital, and training on LPM were the factors associated with good knowledge of SBAs on LPM in the bivariate logistic regression analysis at *p* value of ≤0.2. On the multivariable logistic regression analysis, age, level of education, and type of hospital were found to have an independent association with knowledge of SBAs about LPM at a *p* value of <0.05.

SBAs ≥31 years old were 5.43 times more likely to have good knowledge about LPM than those ≤30 years old (AOR = 5.43; 95% CI: 1.25, 23.53). SBAs with educational level of second degree and above were 3.56 times more likely to have good knowledge on LPM than those with first degree and below level of education (AOR = 3.56; 95% CI: 1.32, 9.60). Compared to those SBAs who were working at public primary hospitals, those who were working at private primary hospitals were 6.55 times more likely to have good knowledge on LPM (AOR = 6.55; 95% CI: 2.15, 19.93). Similarly, those who were working at public referral hospitals were 2.24 time more likely to have good knowledge on LPM than those working at public primary hospitals (AOR = 2.24; 95% CI: 1.01, 4.93) Table (3).

### 3.6. Factors Associated with Attitude of SBAs towards LPM

On bivariate logistic regression analysis, working institution and status of knowledge on LPM were variables associated with attitude of SBAs towards LPM at a *p* value of ≤0.2.

On the multivariable logistic regression analysis, only status of knowledge on LPM showed an independent association with attitude of SBA towards LPM at a *p* value < 0.05. Thus, SBAs with a good status of knowledge on LPM were 2.26 times more likely to have a good attitude towards LPM than their counterparts (AOR = 2.26; 95% CI: 1.42, 3.60) (Table 4).

### 3.7. Factors Associated with SBAs' Practice of LPM

Educational status, monthly salary, type of hospital, and knowledge on LPRMs were the factors associated with SBAs' practice of LPM on the bivariate logistic regression analysis at a *p* value of ≤0.2. On a multivariable logistic regression analysis, type of hospital is the only factor which has an independent association with SBAs' practice on LPM at a *p* value of <0.05. Accordingly, SBAs working at private primary hospitals were about 7 times more likely to practice LPM as compared to those SBAs who were working at public primary hospitals (AOR = 7.01; 95% CI: 1.92, 25.65) (Table 5).

## 4. Discussion

In the current study, about 47.02% of the SBAs had good knowledge on LPM. This result is lower than what was reported in a study conducted in India (87%) [14]. This could be attributable to the difference in the study population, where only obstetricians were included in the study conducted in India. The finding was also lower than a magnitude from a cross-sectional study conducted in three public hospitals found in rural and urban areas of Ethiopia (79%) [15]. This discrepancy could be due to a difference in the tool used to assess knowledge status, where they assessed knowledge using a single indicator. Similarly, another cross-sectional study conducted in Tigray region of northern Ethiopia shows that about 60.1% of the respondents had good knowledge, which was greater than the result of the current study [12]. This could be as a result of difference in study area, where only general hospitals were included in their study.

However, the result of this study is higher as compared to a study conducted in Saudi Arabia where 38.1% of the SBAs had good knowledge [16]. This could be attributed to difference in the study setting and area. Compared to a study conducted in Nigeria, the finding of this study was found to be greater, where 33.3% of the respondents had good knowledge on their study [17]. This discrepancy could arise due to difference in the study population where only nurses were included in their study.

Respondents aged 31 years and above were 5.43 times more likely to have good knowledge about LPM than those SBAs aged 30 years and below. This finding is consistent with a study conducted at United States of America [18]. This could be due to the association of knowledge with development and maturity level; advancing in age will increase the maturity level and responsibility of a working person. In the current study, even if the people older than 31 are only 4% of the overall participants and results regarding age and knowledge must be taken with caution, this data might highlight a gap in the education of young SBAs that must be addressed. In addition, the association could be ascribed to limitations of data collection method, self-report; the reason for this finding might be attributed to the fact that younger SBAs have a poor perception of their knowledge while older SBAs feel more self-confident. Therefore, further studies are recommended to be conducted by taking the aforesaid limitation into consideration. Those SBAs with educational level of second degree and above were 6.55 times more likely to have good knowledge on LPM than those with first degree and below level of education. This finding is in agreement with previous studies which showed that knowledge regarding LPM was determined by the level of education of SBAs [12,18]. This could be due to the fact that a skill in searching and acquiring for new knowledge increases as the level of education of the person increases. This implies that ensuring continuous professional development (CPD) of the SBAs would be helpful in enhancing knowledge on LPM. However, the finding needs to be interpreted cautiously as the result is generated from the data which were gathered through self-report from the SBAs and only 8.9 % of the SBAs were ≥2^nd^ degree holders.

Those SBAs who were working at private primary hospitals were 6.55 times more likely to have good knowledge about LPRMs as compared to SBAs working at public primary hospitals. Similarly, those who were working at the public referral hospitals were 2.24 times more likely to have good knowledge about LPRMs than those working at public primary hospitals. This finding is consistent with findings of the studies undertaken in Saudi Arabia, Nigeria, Australia, and Belgium [16, 19–21]. This could be because of the institutional policies in private sector focusing on giving high standard of care so as to attract clients, and therefore have educated manpower.

Concerning attitude towards LPM, the current study reveals that the proportion of SBAs having a favorable attitude towards LPM is 41.96%. This result is lower compared with a cross-sectional study conducted in India (87%) [12]. This discrepancy could be attributed to the difference in the study population, where only obstetricians were included on the study conducted in India. The finding was also lower as compared to a result reported from a study in Ethiopia (77%) and a study conducted in Nigeria (94.8%) [15, 20]. This could be due to the difference in the tool to assess knowledge status where both of the previous aforementioned studies assessed knowledge using a single indicator. Similarly, another study conducted in northern Ethiopia showed that about 56.7% of the respondents had favorable attitude which was higher compared with the result of the current study [12]. This discrepancy could be due to a difference in the study area where only general hospitals were included in their study.

However, the result of the current study was higher as compared to a study conducted in Amhara Region of Ethiopia where only 26.4% of the SBAs had a favorable attitude towards LPM [22]. This could be due to a difference in the study settings and areas.

Those SBAs who had good knowledge on LPM were 2.26 times more likely to have a favorable attitude LPM towards as compared to SBAs with a poor knowledge about LPRMs. This finding is consistent with a study done in the United States of America [18]. This could be explained as an increase in knowledge and awareness about LPM will make the SBAs tend to have a good attitude towards LPM.

In this study, the proportion of SBAs having a good practice of LPM is 57.14%. The finding is in line with a cross-sectional study done in public hospitals of Addis Ababa, Ethiopia, where more than half (54.2%) of the study participants had a good LPM practice [23].

However, the practice of LPM of this study was found to be greater as compared to a study conducted in Ibana, Nigeria (34.5%) [24]. This discrepancy could be because of the difference in the study population in which only nurses and midwives were included in their study. Similarly, the finding of the current study was higher compared with a study conducted in public health facilities of Kembata Tembaro zone, Ethiopia, where only 37.9% of the respondents had a good LPM practice [25]. This discrepancy in magnitude of LPM practice could be ascribed to variation in the study settings in which health centers were also included in their study. On the contrary, the result of a current study is lower than a magnitude reported from a study done in Egypt where about 63.2% of the study participants had a good LPM practice [13]. The discrepancy could arise from a difference between the study areas.

Those SBAs who were working at private primary hospitals were about 7 times more likely to practice LPM as compared to those SBAs working at public primary hospitals This finding is consistent with findings of two cross-sectional studies conducted in Ethiopia [23, 25]. The association could be because of the institutional policies in the private sectors which focus on giving high standard of care so as to attract clients, and therefore have better trained manpower to provide LPM.

Finally, the authors would like to advise the readers to interpret the findings of this study with caution as it has certain limitations. The reports of this study might be overestimated as the source of data for this study was based on the self-report of respondents which is prone to recall bias. However, with all those limitations, we strongly believe that this study is helpful to the scientific community as it was conducted in the settings where there was limited evidence. Thus, it will provide a baseline insight for researches, thereby inspiring future researches in this theme.

## 5. Conclusion

In this study, the proportion of SBAs having good knowledge, a favorable attitude, and a good practice of LPM was lower than the results of many of the previous studies. Strengthening the capacity of the public health facilities and assuring continuous professional development (CPD) through trainings specific to LPM would be helpful in improving LPM practices.

## Figures and Tables

**Table 1:** Sociodemographic characteristics of SBAs working at hospitals in Gondar zones, northwest Ethiopia, 2019.

Variables		Frequency (%)
Sex	Male	195 (58.0%)
	Female	141(42.0%)

Age (years)	≤30	321 (95.5%)
	≥31	15 (4.5%)

Religion	Orthodox	215 (64.0%)
	Muslim	84 (25.0%)
	Protestant	37 (11.0%)

Marital status	Never married	159 (47.3%)
	Married	171 (50.9%)
	Divorced	6 (1.8%)

Profession	Nurse	45 (13.4%)
	Midwifery	188 (56.0%)
	Emergency surgeon	20 (6.0%)
	General practitioner	71 (21.1%)
	Gynecologist	12 (3.6%)

Level of education	Diploma	4 (1.2%)
	1^st^ degree	302 (89.9%)
	2^nd^ degree and above	30 (8.9%)

Monthly salary (per birr)	3553–7000	234 (69.6%)
	7001–10000	61 (18.2%)
	>10000	41 (12.2%)

Working institution	Public primary hospital	276 (82.1%)
	Private primary hospital	24 (7.1%)
	Referral hospital	36 (10.7%)

Availability of at least one labor analgesia drug	Yes	331 (98.5%)
	No	5 (1.5%)

**Table 2:** Knowledge of SBAs on nonpharmacologic labor pain relief methods who are working at hospitals found in Gondar zones, northwest Ethiopia, 2019.

Variables		Frequency (%)
Psychotherapy	Yes	183 (54.5%)
	No	153 (45.5%)

Allow the mother to ambulate	Yes	163 (48.5%)
	No	173 (51.5%)

Massage the back	Yes	148 (44.0%)
	No	188 (56.0%)

Allow free vertical positioning	Yes	113 (33.6%)
	No	223 (66.4%)

Transcutaneous electrical nerve stimulation	Yes	62 (18.5%)
	No	274 (81.5%)

Show the woman how to bear down	Yes	109 (32.4%)
	No	227 (67.6%)

Acupuncture	Yes	11 (3.3%)
	No	325 (96.7%)

Hypnosis	Yes	30 (8.9%)
	No	306 (91.1%)

Allow companion of laboring woman of choice	Yes	9 (2.7%)
	No	327 (97.3%)

Music therapy	Yes	3 (0.9%)
	No	333 (99.1%)

**Table 3:** Factors associated with knowledge on LPM among SBAs working at hospitals in Gondar zones, northwest Ethiopia, 2019.

Variables		Knowledge on LPM		COR (95% CI)	AOR (95% CI)
		Good	Poor		
Sex	Male	102	93	1.67 (1.07–2.58)	
	Female	56	85	1	

Age	≤30	155	166	3.74 (1.03–13.49)	5.43 (1.25–23.53)^*∗*^
	≥31	3	12	1	

Educational status	≤1^st^ degree	137	169	1	
	≥2^nd^ degree	21	9	2.88 (1.28–6.49)	3.56 (1.32–9.60)^*∗*^

Hospital type	Public primary	113	163	1	
	Private primary	20	4	7.21 (2.4–21.67)	6.55 (2.15–19.93)^*∗*^
	Referral	25	11	3.28 (1.55–6.93)	2.24 (1.01–4.93)^*∗*^

Got training on LPM	Yes	119	108	2.03 (1.27–3.26)	
	No	38	70	1^*∗*^	

1 = reference category, ^*∗*^ =  *p* value <0.05.

**Table 4:** Factors associated with attitude on LPM among SBAs working at hospitals in Gondar zones, northwest Ethiopia, 2019.

Variables		Attitude on LPM		COR with 95% CI	AOR with 95% CI
		Favorable	Unfavorable		
Type of the hospital	Public primary	126	150	1^*∗*^	
	Private primary	1	23	0.05(0.01–0.39)	
	Referral	14	22	0.76(0.37, 1.54)	

Knowledge status on LPM	Good	77	81	1.69(1.09–2.62)	2.26 (1.42, 3.60)^*∗∗*^
	Poor	64	114	1^*∗*^	

1^*∗*^ = reference category, ^*∗∗*^ = *p* value <0.05.

**Table 5:** Factors associated with practice on LPM among SBAs working at hospitals in Gondar zones, northwest Ethiopia, 2019.

Variables		SBA's practice on LPM		COR (95% CI)	AOR (95% CI)
		Good	Poor		
Educational status	≤1^st^ degree	168	138	1^*∗*^	
	≥2^nd^ degree	24	6	3.29 (1.31–8.27)	

Monthly salary (birr)	≤6055	110	117	1^*∗*^	
	>6055	82	27	3.23 (1.95–5.36)	

Type of the hospital	Public primary	147	129	1^*∗*^	
	Private primary	21	3	6.14 (1.79–21.07)	7.01 (1.92, 25.65)^*∗∗*^
	Referral	24	12	1.76 (0.84–3.65)	

Knowledge on LPM	Good	103	55	1.87 (1.21–2.91)	
	Poor	89	89	1^*∗*^	

1^*∗*^ = reference category, ^*∗∗*^ = *p* value <0.05.

## Data Availability

The authors declare that the data regarding this manuscript can be accessed as per the request of any interested body and can be accessed by emailing the corresponding author using on fissehayetwale99@gmail.com.

## Abbreviations

AOR: Adjusted odds ratio
COR: Crude odds ratio
CI: Confidence interval
EFMOH: Ethiopian Federal Ministry of Health
EPI INFO: Statistical Package for Epidemiological Information Analysis
ETB: Ethiopian birr
KAP: Knowledge, attitude, and practice
LPM: Labor pain management
LPRMs: Labor pain relief methods
SBAs: Skilled birth attendants
SD: Standard deviation
SPSS: Statistical Package for Social Sciences.

## Supplementary Materials

Supplementary Materials Additional file 1 is a questionnaire comprising questions on participants' sociodemographic characteristics; knowledge, attitude, and practice on labor pain management; reasons for not practicing labor pain management; and institutional factors which have been adapted from previous similar literature [13].
