# Supplementary material for: Knowledge, Attitude, and Practice towards Labor Pain Management and Associated Factors among Skilled Birth Attendants Working at Hospitals Found in Central, West, and North Gondar Zones, Northwest Ethiopia, 2019: A Multicenter Cross-Sectional Study
Source: Pain Res Manag. 2021 May 13;2021:8835677. doi: 10.1155/2021/8835677 (PMC8140842; doi:10.1155/2021/8835677)
Supplement: Supplementary Materials — Additional file 1 is a questionnaire comprising questions on participants' sociodemographic characteristics; knowledge, attitude, and practice on labor pain management; reasons for not practicing labor pain management; and institutional factors which have been adapted from previous similar literature [13]. [file 8835677.f1.docx]

Additional file 1: Questionnaire

| **Section I: Socio- Demographic Characteristics**  **Instructions:** Please complete the following items regarding your demographics information by encircling or fill in the response/ statement that is closest to what best describes you. | |
| --- | --- |
| 101. Your institution/hospital | _________________________ |
| 102. Age of respondent | _________ years |
| 103. Sex | 1. Male  2. Female |
| 104. Your monthly income | _____________Ethiopian birr |
| 105. Religion | 1. Orthodox 2. Protestant  3. Muslim 4. Other specify _______________ |
| 106. Marital status | 1. Never married 2. Married  3. Divorced 4. Widowed |
| 107. Level of education | 1. Diploma 2. First degree  3. Second Degree and Above |
| 108. Profession : | 1. Nurse  2. Midwife  3. Health Officer  4. Emergency Surgeon  5. General Practitioner  6. Gynecologist |
| 109. Years of working experiences | ___________( years) |
| 110. Does equipment and analgesics drugs for pain management are available in your institution? | 1. Yes 2. No |
| 111. Have you ever take in-service training about labour pain management? | 1. Yes 2. No |

| **II. Knowledge Related Questions** | | | |
| --- | --- | --- | --- |
| 201 | Do you know about labor pain management methods? | 1. Yes  2. No | **If “No”, please skip to Q No 208** |
| 202 | If “Yes” is your response to question No 201, what type of labor pain management do you know? (More than one answer is possible….) | 1. Pharmacologic  2. Non-pharmacologic  3. Both  4. Not sure | **If “2”, please skip to Q No 204** |
| 203 | If “Pharmacological” is your response to question No 202, which pharmacologic method do you know? (More than one answer is possible….) | 1. Systemic opioids  2. NASID drugs  3. Epidural analgesia  4. Inhalational  5. If other, please specify______________________________________________________ |  |
| 204 | If “non- pharmacological” is your response to question No 202, which type of non-pharmacologic method, do you know? (More than one answer is possible…..) | 1. Psychotherapy  2. Allow the mother to ambulate  3. Massage the back  4. Allow free vertical positioning  5. Trans cutaneous electrical nerve stimulation  6. Show the patient how to bear down  7. Acupuncture  8. Hypnosis  9. Allow companion of her choice  10. Music therapy  11. If other, please specify______________________________________________________ |  |
| 205 | If you know about labour analgesia, do labour relief method have side effect on labour and delivery outcome? | 1. Yes  2. No  3. I don’t know | **If “2” & “3”, please skip to Q No 207** |
| 206 | If” yes” to question 205, what is the side effect of analgesia on labour and its outcome? (More than one answer is possible…..) | 1. Delay progress of labour  2. Cause fetal distress  3. Increase instrumental delivery  4. Increase C/S delivery  5. Chronic back pain  6. If other, please specify______________________________________________________ |  |
| 207 | Have you ever asked labouring women to provide labour pain relief method? | 1. Yes  2. No  3. I don’t remember |  |
| 208 | Have you heard about WHO pain ladder? | 1. Yes  2. No  3. Unsure |  |
| **III. Attitude Related Questions** | | | |
| 301 | Do you believe that labour pain management methods can alleviate or help the mother to cope labour pain? | 1. Yes  2. No |  |
| 302 | Do you think that every mother during labour should be managed? | 1. Yes  2. No |  |
| 303 | Do you believe that a mother hasn't to face labor pain even though it is natural? | 1. Yes  2. No |  |
| 304 | Do you think analgesic is necessary for managing labour pain? | 1. Yes  2. No |  |
| 305 | Do you believe that you have responsibility and obligation to manage labour pain? | 1. Yes  2. No |  |
| 306 | Would you provide pain relief if you had resources? | 1. yes  2.No  3.Unsure |  |

| **IV. practice of labour pain relief method** | | | |  |
| --- | --- | --- | --- | --- |
| 401 | Have you ever provided any labour pain relief method in the past one month? | 1. Yes 2. No 3. Don’t remember | **If “2” & “3”, please skip to Q No 501** | |
| 402 | If “Yes” is your response to question No 401, which method did you provide? | 1. Pharmacological 2. Non-pharmacological   3.Both | **If “2”, please skip to Q No 404** | |
| 403 | If “Pharmacological” is your response for question number 402, which method did you provide? (You can answer more than one….) | 1. Pethidine 2. Diclofenac 3. Paracetamol 4. Hyoscine 5. If other, please specify________________ |  | |
| 404 | If “non-pharmacological” is your response for question number 402, which type non pharmacologic did you provide? (You can answer more than one.....) | 1. Psychotherapy 2. Allow the mother to ambulate 3. Massage the back 4. Allow free vertical positioning 4. Show the patient how to bear down 5. Allow companion of her choice 6. Hot compress 7. Music therapy 8. If other, please specify___________________ |  | |
| **V. Questions on Reasons for Non-use of Labour Pain Management Methods** | | | |  |
| 501 | Reasons for non-practical of labour pain management methods?  Multiple options are possible…. | 1. High patient flow 2. Lack of knowledge &   skill   1. No drug 2. No equipment 3. Small number of staff 4. If other, please specify________________ |  | |
| **VI. Questions Related to Institutional Factors** | | | |  |
| 601 | If you know pharmacologic methods, are labour pain managing drugs/ analgesics available at your hospital? | 1. Yes 2. No 3. I don’t know | **If “2” & “3”, please Skip to Q No 603** | |
| 602 | If “Yes” to Question No 601, which type is available? (You can answer more than one….) | 1. Pethidine 2. Diclofenac 3. Paracetamol 4. Hyoscine 5. If other, please specify_________________ |  | |
| 603 | Have you got any training for managing labour pain? | 1. Yes 2. No |  | |
| 604 | Does your public center allow companion of choice for a labouring mother? | 1. Yes 2. No |  | |
| 605 | Do you allow a woman to move around while she is in labour? | 1. yes 2. No |  | |
